# Phylogeography of the *Lutzomyia gomezi* (Diptera: Phlebotominae) on the Panama Isthmus

**DOI:** 10.1186/1756-3305-7-9

**Published:** 2014-01-08

**Authors:** Anayansi Valderrama, Mara Garcia Tavares, Jose Dilermando Andrade Filho

**Affiliations:** 1 Department of Medical Entomology, Instituto Conmemorativo Gorgas de Estudios de la Salud, Panama, Panama; 2 Department of Biology, Universidade Federal de Viçosa, Viçosa, MG, Brasil; 3 Centro de Referência Nacional e Internacional para Flebotomíneos/Coleção de Flebotomíneos, Instituto René Rachou-Fiocruz, Belo Horizonte, MG, Brasil

**Keywords:** Panamá, Sandflies, *Leishmania panamensis*, Cutaneous leishmaniasis, Genetic variability and phylogeographic

## Abstract

**Background:**

*Lutzomyia gomezi* (Nitzulescu, 1931) is one of the main *Leishmania* (*Vianna*) *panamensis* vectors in Panama, and despite its medical significance, there are no population genetic studies regarding this species. In this study, we used the sequences of the mitochondrial gene cytochrome b/start of NADH1 and the nuclear elongation gene α-1 in order to analyze genetic variation and phylogeographic structure of the *Lu. gomezi* populations.

**Methods:**

A total of 86 *Lu. gomezi* individuals were captured in 38 locations where cutaneous leishmaniasis occurred. DNA was extracted with phenol/chloroform methods and amplification of genes was performed using PCR primers for mitochondrial and nuclear markers.

**Results:**

We found a total of 37 and 26 haplotypes of mitochondrial and nuclear genes, high haplotype diversity (h) for all three populations were detected with both molecular markers. Nucleotide diversity (π) was estimated to be high for all three populations with the mitochondrial marker, which was opposite to the estimate with the nuclear marker. In the AMOVA Φst recorded moderate (mitochondrial) and small (nuclear) population structure with statistical significance among populations. The analysis of the fixation index (Fst) used to measure the differentiation of populations showed that with the exception of the population located in the region of Bocas del Toro, the other populations presented with minor genetic differentiation. The median-Joining network of the mitochondrial marker reveled three clusters and recorded four haplotypes exclusively of localities sampled from Western Panama, demonstrating strong divergence. We found demographic population expansion with Fu´s Fs neutrality test. In the analysis mismatch distribution was observed as a bimodal curve.

**Conclusion:**

*Lu. gomezi* is a species with higher genetic pool or variability and mild population structure, due to possible capacity migration and local adaptation to environmental changes or colonization potential. Thus, knowledge of the genetic population and evolutionary history is useful to understand the implications of different population genetic structures for cutaneous leishmaniasis epidemiology.

## Background

Cutaneous leishmaniasis is the most common form of leishmaniasis reported in the Republic of Panama; its clinical manifestations range from minor lesions to severe skin ulcers [1-3]. In Panama it was first recorded in 1910 [4], an overall of 15 cases occurred during 1910–1944; but there has been a sharp increase since 2000 [5]. According to the Epidemiology Department of the Health Ministry of Panama, a total of 26,163 cases of cutaneous leishmaniasis occurred during 2000–2010 and the distribution of occurrence of CL was in regions of the provinces of Bocas de Toro (28%), Panama West (20%), Coclé (17%), Colón (11%), Panama East (5%), Veraguas (3%). The disease is mainly caused by *Leishmania* (*Vianna*) *panamensis* and *Lutzomyia ylephiletor*, *Lu. sanguinaria*, *Lu. panamensis*, *Lu. trapidoi*, *Lu. gomezi* were identified as the transmission vectors [6-8].

In Panama, recent research has show that *Lutzomyia* (*Lutzomyia*) *gomezi* (Nitzulescu, 1931) is the most abundant species with wide geographical distribution and their abundance has been associated with cases of clinical CL acquired in households of rural communities [9]. Alongside *Lu. gomezi* other *Lutzomyia* species vectors have been caught in the same place and associated with a focus of cutaneous leishmaniasis infection in other American countries, incriminating this species with high potential in the transmission of the disease [2,10-14].

*Lutzomyia* (*Lutzomia*) *gomezi* in Central America is present from Mexico to Panama and Trinidad Tobago (Caribbean), also recently reported in Guatemala [14]. In South America, it has been reported in Colombia, Venezuela, Ecuador, Peru, French Guiana and Brazil, specifically in Amapá, Acre, Pará, Mata Grosso, Goiás, Bahia, Maranhão and Rondônia [15-17].

In general, its natural environment is a humid and dark place, such as nests, rock crevices, animal burrows and tree bark in the tropical rainforest [2]. However, it is also reported in forest gaps and the canopies were light availability produces humid change [2,18]. The foraging and blood feeding behavior occurs at twilight and at night from 18:00–20:00 hours [18]. Nevertheless, the deforestation and loss of natural habitat have caused its adaptation to peridomestic areas, perhaps changing the hourly activity and feeding on a large variety of domestic animals [19,20]. In the focus of leishmaniasis, this species is predominant near peridomestic or outdoor areas more than indoors households, which is difficult to control [2,19,21-23].

Despite of the potential significance of *Lu. gomezi* as a *Leishmania* vector, few researches have targeted genetic aspects or interrelationship of host-vector species. For instance, [24] detected a natural infection of *Lu. gomezi* with the *Le. braziliensis* in Venezuela, in order to establish a methods for determine the circulation of *Leishmania* parasites in leishmaniasis endemics areas. On the other hand, [25] analyzed the changes to the primary and secondary structures of tRNA_ser_ of the species; *Lu. trinidadensis* (Oswaldoi group), *Lu*. (Psychodopygus) *panamensis*, *Lu*. (*Micropygomyia*) *cayennensis cayennensis*, *Lu. dubitans* (Migonei group), *Lu*. (*Lutzomyia*) *gomezi*, *Lu. rangeliana* (ungrouped) and *Lu. evansi* (Verrucarum group) for taxonomic purposes, considering that morphological identification can be difficult. Also, [26] detected with ITS-1 a pool of *Lu. gomezi* infected with *Le. naiffi* in Panama, the first report for the country which prompted several hypotheses on the introduction of this parasite into this country.

Attempts to understand the role that arthropod vectors play in disease dynamics and pathogen transmission of leishmaniasis, several studies over genetic population of sandfly vectors has been performance in the Latin America, Iran, Turkey, Palestine, Israel and Egypt [27-30]. Many of these studies are focused in two principal species, *Lu. longipalpis* and *Phlebotomus papatasi* to determinate their genetic variation, structures and differentiation of populations [27,28,31,32]. Thereby, molecular evidence for divergence of vectors has been found and an assessment of the impact on leishmaniasis epidemiology. For instance, the taxonomic status of the *Lu. longipalpis* complex has been fundamental to the understanding of leishmaniasis epidemiology. Symptoms observed for transmission of *Leishmania infantum chagasi* by *Lu. longipalpis* in Brazil and Colombia results in visceral infections, whereas the transmission of the same parasite by *Lu. longipalpis* in Costa Rica results in non-ulcerative lesions [33,34].

Thus, it becomes important to analyze the population genetics and demographic history of this vector species to assess the genetic pool, gene flow and colonization potential, consequently known as the population changes and genetic variation affecting the vector competence and resistance to insecticides [35,36]. On the other hand, the population genetics analysis can provide information about migration capacity, reproduction, and adaptation to the conditions of the new habitat, favoring the emergence of vector diseases [37]. In addition to this, the determination of cryptic vector species and their ability to transmit pathogens are even more relevant to understand their implications in the epidemiology of vector diseases and to suggest appropriate and effective prevention and control programs without any environmental risks [36].

Due to the lack of population genetics information of *Lu. gomezi* species in Panama and the Americas as a whole, the goal of this research was to evaluate and compare the intra- and inter-population variability of *Lu. gomezi* from localities with a high incidence of cutaneous leishmaniasis in Panama. Also to identify the barriers that may influence gene flow among *Lu. gomezi* populations. Moreover, to infer the historical processes that defines actual geographic distributions of the species. These results may contribute on the knowledge of leishmaniasis epidemiology and improve the development of focal or large-scale programs control for leishmaniasis vectors in Panama.

## Methods

### Study sites

The study was performed in the Republic of Panama between the coordinates 7º11′-9°39′N and 77º10′-83º03′W. The most prevalent climatic regime in Panama is tropical humid, with the dry season (January-March) presenting an average temperature of 31.5ºC and relative humidity of 75%. In other months (April-December), the average temperature is 27ºC and the relative humidity averages 90%. The vegetation in Panama varies according to climate zones and consists mainly of tropical humid forest or savannahs resulting from agricultural activity.

### Sandfly collections and identification

**Figure 1:**
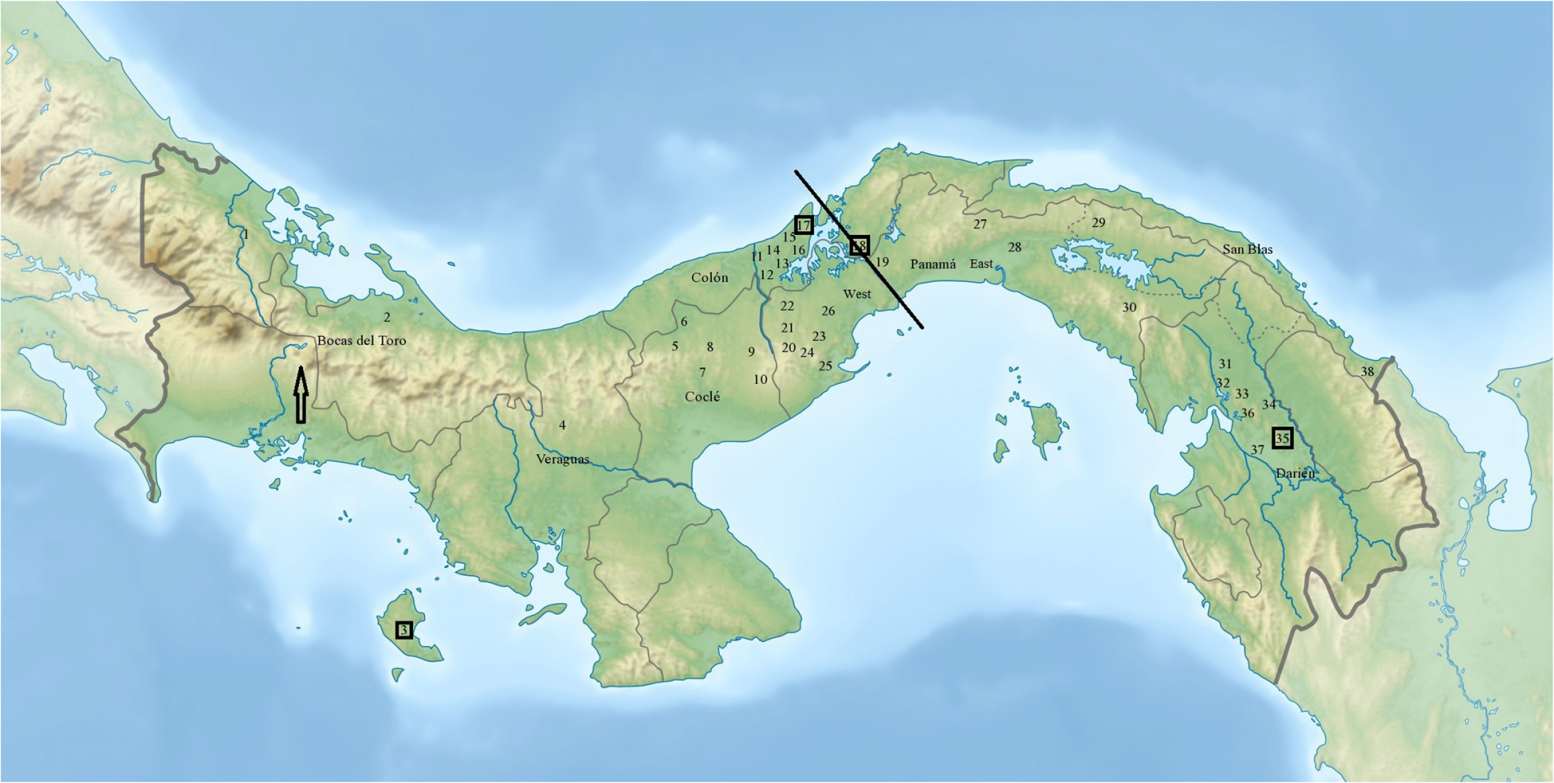
**Map of the Panama isthmus and distribution of the sampling sites of *Lutzomyia gomezi*.** The arrow indicates the Cordillera Central and line (−) represents the Panama Canal. The numbers inside the square symbolize four natural reserves (forest) protected by the National Environmental Authority (ANAM), used as control.

*Lu. gomezi* samples were collected from the thirty-eight localities in Panama, during the dry season (January-April) and rainy season (May-June) of 2010. Thirty-four sites were chosen, representing incidence with cases of cutaneous leishmaniasis in humans during the 2006–2009 period, according to data from the Department of Epidemiology of the Ministry of Health of Panama; four sampling sites were in Panamanian tropical forests. The geographic distribution of the samples locations are indicated in Figure 1.

The characteristic of the thirty-four collecting sites according to the landscape was fragmented environment (secondary vegetation and areas used for agricultural activities) or rural (an area without a large concentration of people). The Coiba National Park, San Lorenzo Park, Oil Pipeline Road Park, and the Serrania Filo del Tallo Park represent natural reserves (forests) protected by the National Environment Authority of Panama (ANAM). The samples localities are listed in Table 1.

**Table 1:** List of sampling sites with information on landscape features, CB3-N1N and EF α-1 haplotypes identified in each location

**Populations**	**Locations**	**Coordinates**		**MacroHabitat**	**Micro Habitat**	**CB3-NIN haplotype**	**EF α-1 haplotype**
Western populations	1-Nance Valle Risco	9.255	−82.470	Fr	P	H1-H2	H1-H2
	2-Bisira	8.899	−81.862	R	P	H3-H4	H1
	3-Parque Nacional Coiba	7.627	−81.730	Fo	S	H6	H4
	4-Altos de Piedra	8.515	−81.087	Fr	P	H5	H1-H3
Central populations	5-Villa del Carmen	8.800	−80.552	R	P	H7	H1
	6-Coclesito	8.811	−80.550	R	P	H9-H11	H1
	7-Molejón	8.767	−80.513	Fr	P	H8-H9-H10	H1-H12
	8-Cutevilla	8.772	−80.487	Fr	P	H10-H11-H12	H1-H24
	9-Vaquilla	8.699	−80.195	Fr	P	H10-H12	H1-H23
	10-Chirigui Arriba	8.663	−80.187	R	P	H13-H14	H3-H22
	11-Quebrada Leona	9.177	−80.139	Fr	P	H15-H16-H17	H1-H17-H18-H19
	12-Cuipo	9.090	−80.051	R	P	H10-H15-H22-H23	H1-H6-H9-H20-H21
	13-Providencia	9.207	−79.999	R	P	H9-H15-H21	H1-H9
	14-Achiote	9.226	−80.030	R	P	H12-H19-H20	H1
	15-Piña	9.244	−80.040	Fr	P	H10-H18	H1
	16-Unión Piña	9.273	−80.021	Fr	P	H10-H15-H22-H23	H1
	17-Parque Nacional San Lorenzo	9.351	−79.973	Fo	S	H10	H1
	18-Parque Nacional Camino del Oleoducto	9.119	−79.700	Fo	S	H15	H1
	19-Altos de Divisa	9.119	−79.693	R	P	H10	H1
	20-Cacao	8.748	−80.017	Fr	P	H10-H28	H1-H5
	21-Trinidad	8.808	−80.019	Fr	P	H29	H1
	22-Vista Alegre	8.808	−80.014	Fr	P	H15-H23	H1-H11
	23-Valdeza	8.789	−79.965	R	P	H10-H27	H1-H9-H10
	24-Caimito	8.739	−79.937	R	P	H23-H29	H6
	25-Limón	8.697	−79.904	Fr	P	H24-H25-H26	H1-H7-H8
	26-Ollas Arriba	8.804	−79.912	R	P	H10	H1-H9-H12
Eastern populations	27-Madroño	9.284	−79.134	Fr	P	H10	H14
	28-Gato Real	9.267	−79.119	Fr	P	H15-H18-H30	H1-H14-H15-H16
	29-Buenos Aires (Chepo)	9.239	−78.817	Fr	P	H10-H15	H1
	30-Torti	8.981	−78.571	Fr	P	H10-H15-H31	H1-H13-H14
	31-Arimay	8.694	−78.146	R	P	H15	H1
	32-La Cantera	8.640	−78.169	R	P	H15-H32	H1-H13-H14
	33-Nicanor	8.544	−78.035	Fr	P	H10-H33-H34	H1-H13
	34-Buenos Aires (Darién)	8.530	−77.961	R	P	H10-H15	H1-H8-H9
	35-Parque Serranía Filo del Tallo	8.465	−77.993	Fo	S	H10	H1
	36-Bijagual	8.459	−78.012	Fr	P	H15-H23-H35	H1-H13
	37-Rio Iglesia	8.401	−78.007	R	P	H15-H36-H37	H8-H14-H25-H26
	38-Puerto Obaldía	8.669	−77.429	Fr	P	H15	H1

Fr: fragmented forest; R: rural; Fo: forest; P: peridomestic; S: selvatic.

Specimens of *Lu. gomezi* used in the study were caught using CDC light traps and octenol solution utilized to attract hematophagous insects [38]. The traps were positioned in the fragmented and rural locations, near peridomestic areas, with the presence of domestic animal (dogs, chickens, pigs, and cats) and ornamental vegetation. In the forest, traps were placed next of stone crevices and tree barks.

A total of nine CDC light traps were exposed at a height of 1.5 m in each locality and were installed at approximately 50 m intervals. The collection period was of 12 h (18:00 h-06:00 h) for two consecutive days at each point. The specimens were sacrificed with chloroform and stored in 95% ethanol at −20°C. Adult *Lu. gomezi* were identified by morphology and genital structure using the identification keys of [16].

### DNA extraction and amplification

The DNA of 86 individual sandflies was isolated by a standard phenol/chloroform technique, precipitated with ethanol and resuspended in Tris-EDTA (TE) buffer according to [39], with some changes and stored at −20°C until used.

Two mitochondrial and nuclear genes were analyzed to estimate the structure and geographic genetics within and among populations of *Lu. gomezi*. The region of mitochondrial genes analyzed was final Cytb gene, inter-genetic region IGS-1, tRNA-Ser, inter-genetic region IGS-2, and start of the NADH1 (CB3-NIN), and amplified using primers CB3-PDR: 5′CAYATTCAACCWGAATGATA3′/N1N-PDR:5′GGCAYWTTGCCTCGAWTTCGWTATGA3′ [27]. Nuclear region elongation factor alpha-1 (EF α-1) coding an intron region were amplified with primers EF-F03 5′CCTGGACATCGTGATTTCAT3′/EF-R04 5′AGTGCTTCGTGGTGTAT(C/T)TC3′ [40].

PCR amplification of genes were performed in a volume of 25 μl containing 1× buffer, 1.5 mM MgCl2^++^, 0.2 mM of each dNTPs, 0.4 μM of each primer, 1.5 units of Taq polymerase and approximately 2 μl of DNA (≈50 ng/μl) template. The details of thermal cycling conditions were outlined by [27] for CB3-N1N and [41] for EF α-1. A negative control was included in each PCR reaction and visualization of PCR product using 1.5% agarose gel with a 100 bp ladder.

### Sequencing and alignment

PCR products were purified and sequenced directly by the Macrogen INC., Seoul, Korea Sequencing Service in both directions with the same primers used in the amplifications. The sequences were compared to the nucleotide BLAST (Basic Local Alignment Search Tool) tool available in GenBank NCBI (National Center for Biotechnology Information) in order to verify the similarities between the sequences of *Lu. gomezi* stored in the database. The CodonCode Aligner software was used to edit the nucleotide sequences and identify the heterozygous as double peaks on the chromatograms of the nuclear region. The sequences were aligned with the Muscle option included in the MEGA 5.05 software [42]. The polymorphic sites, single substitution (singleton) and average base frequencies were computed with MEGA 5.05 software.

### Data analysis

To carry out an analysis of population genetics (genetic diversity, structure and differentiation) to specimens collected in different localities, we clustered and defined samples in western, central and eastern populations from Panama (Table 1). Thus, the specimens collected in Bocas del Toro, Veraguas and Coiba represented the western populations, those collected from Coclé, Colon, and west of the Panama canal were central populations, whereas specimens from east of the Panama canal, Darién and San Blas corresponding to the eastern population (Figure 1).

### Genetic diversity

We emphasized that heterozygotes detected in the EF α-1 were treated with recombination model in PHASE option, incorporated in the DnaSP v.5. To estimate the genetic diversity we used the following indexes: haplotypes number, haplotype diversity (h), and nucleotide diversity (π) for each population. DnaSP v.5 [43] and Arlequin 3.11 [44] software was used for this purpose.

### Genetic structure and differentiation

The population structure was determined performing an Analysis of Molecular Variance (AMOVA) [45,46]. The Arlequin 3.11 [44] software analyzed the variance components and significance levels p = 0.01 using non-parametric permutations of 1,000 times. To estimate levels of genetic differentiation among the populations a pairwise comparison test was performed. Non-parametric permutations of 1,000 times and a significance p = 0.01 was used to estimate Fst-value [45] in Arlequin 3.11 [44] software.

### Network haplotypes

A haplotype network for both genes was inferred through the median-joining network method [47], using the Network v.4.5 software. In order to obtain the values of posteriori probabilities among the clades, a Bayesian analysis was calculated using MrBayes 3.1 [48] program. MrModelTest [49] software was used to choose the best model of nucleotide substitution according to the AIC (Akaike Information Criterion). The model obtained by MrModeltest for CB3-N1N Bayesian inference analysis was HKY + I, with the proportion of invariable places (I) = 0.7594. For EF α-1, the model obtained was K80 + I, with a proportion of invariable places (I) = 0.6725. 10 million generations of Markov Chain Monte Carlo (MCMC) and burn-in of 5 million were used.

### Demographic history of population

In addition to this, Tajima’s D [50] was used for estimate deviations from neutrality mutation or selective neutrality of these populations, P-value 0.01 was considered statistically significant. Also Fu’s Fs [51] were calculated for neutrality of mutation against population growth, hitchhiking and background selection, P-value 0.02 as significant. Both values Tajima’s D and Fu’s Fs were calculated using the software Arlequin 3.11 [44] with permutations of 1,000 times.

The mismatch distribution was calculated for CB3-N1N and EF α-1 as supplementary test to evidence of demographic expansion of the population, through sudden expansion model [52]. In Arlequin 3.11 [44] we computed the sum of square deviations (SSD) and Harpending´s raggedness index to test the goodness-of-fit of the model to the data. The time since population expansion was inferred using CB3-NIN region by means of the equation t = τ /2 μ, where tau (τ) was obtained on the mismatch distribution outputs and a mutation rate (μ) of 1.1-1.2% per million years in *Heliconius erato*[53].

## Results

### Sequence characterization

Overall, 86 sequences isolated of CB3-NIN and EF α-1 of *Lu. gomezi* individuals from 38 selected localities from Panama Isthmus were analyzed. Of these, an alignment of 501 nucleotides was obtained of CB3-N1N with thirty-nine polymorphic sites and nineteen single substitutions (singleton). The average base frequencies were A = 37%, C = 15%, G = 10%, and T = 38% recording a sequence rich in A-T (75%). A fragmented size of EF α-1 was 597 bp, in this case 166 sequences were obtained by clone generating with the recombination model algorithm of DnaSP v.5 considering the heterozygous individuals. Of those sequences, thirty-three polymorphic sites, six singletons and a larger proportion of G-C (53%): 23%T, 27%C, 23%A, 27%G, were detected.

### Genetic diversity

For the analysis of genetic diversity indexes both CB3-NIN and EF α-1 region demonstrated high haplotype diversity (h) for all three populations [Table 2]. While the nucleotide diversity (π) for the mitochondrial region was estimated to be high for all three populations, nuclear nucleotide diversity was opposite to the estimate with mitochondrial, showing a low value [Table 2]. The haplotype and the landscape features per collection site are showed in [Table 1].

**Table 2:** **Genetic diversity data obtained with analysis of CB3-NIN and EF α-1of the *Lu. gomezi***

**CB3-NIN**	**Western**	**Central**	**Eastern**
	**populations**	**populations**	**populations**
Nº individual	7*	51	28
Nº haplotype	6	23	12
h(d) ± SD	0.95 ±0.096	0.89 ± 0.033	0.80 ± 0.061
π(d) ± SD	0.01 ± 0.008	0.01 ± 0.005	0.01 ± 0.006
**EF α-1**	**Western**	**Central**	**Eastern**
	**populations**	**populations**	**populations**
Nº individual	7*	51	28
Nº haplotype	4	18	9
h(d) ± SD	0.58 ± 0.137	0.58 ± 0.060	0.63 ± 0.069
π(d) ± SD	0.003 ± 0.002	0.004 ± 0.003	0.003 ± 0.002

H(d): haplotype diversity; π(d): nucleotide diversity; SD: standard deviations.* We highlight that the collecting sites of Bocas del Toro and Veraguas were difficult to access therefore have a much smaller sample size.

### Genetic structure and differentiation

In the AMOVA Φst estimated between the three populations, CB3-N1N region recorded moderate and EF α-1 small population structure with statistical significance among populations. The total genetic variation found within the populations was ≈ 86% (CB3-NIN) and ≈ 95% (EF α-1) as shown in Table 3. Pairwise comparison analysis of the mitochondrial region revealed strong genetic differentiation with highly significance between Western Panama and Central Panama, whereas the Fst-value between Western and Eastern Panama, showed a moderate and significant differentiation. However, when populations were analyzed with nuclear region it revealed weak differentiation among populations of Western Panama and Eastern Panama. Also a moderate level of genetic differentiation was observed among the populations a Central Panama and Eastern Panama [Table 4].

**Table 3:** **Analysis of molecular variance (AMOVA) based on CB3-NIN and EF α-1sequence of the populations of *Lu. gomezi***

**CB3-NIN**					**EF α-1**			
**Source of variation**	**d.f**	**(%)**	**Fixation index**		**d.f**	**(%)**	**Fixation index**	
		**variations**				**variations**		
Among populations	2	13.7	Φ_ST_=	0.13*	2	4.5%	Φ_ST_=	0.04*
Within populations	83	86.3			163	95.5%		

d.f = degrees of freedom; * *P* < 0.01.

All samples were grouped according to Table 1.

**Table 4:** **Estimates of pairwise Fst of CB3-N1N and EF α-1 between *Lu*. *gomezi *** **populations**

**CB3-NIN**	**Western populations**	**Central populations**
**EF α-1**		
Western populations	*	
Central populations	0.23	*
	0.02	
Eastern populations	0.21	0.10
	0.04	0.12

Significance (p < 0.05)*.

### Network haplotype

**Figure 2:**
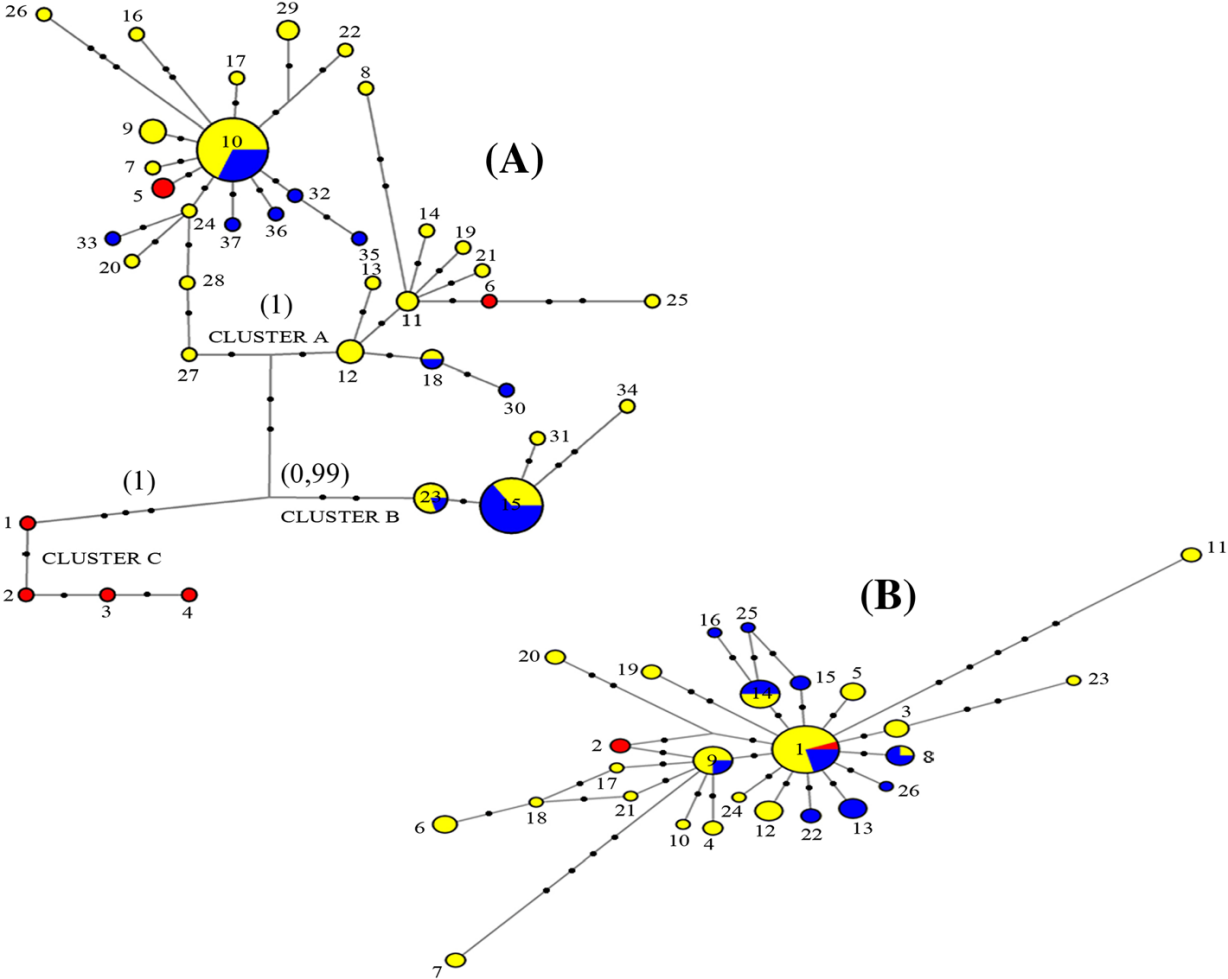
**A haplotype network inferred by a median-joining method using CB3-NIN sequences (A) and EF α-1 sequences (B) of *Lutzomyia gomezi*.** Circles represent different haplotypes and sizes that are proportional to haplotype frequencies. The length of the traces indicate number of mutations among haplotypes. Each color of circles showed the geographical populations: Western (red), Central (yellow), Eastern (blue). Values in parentheses showed the *a posteriori* probability of Bayesian inference.

The median-Joining network reconstructed with CB3-NIN region linked a total of 37 different haplotypes with three defined clusters (A, B, C). The lack of hierarchic structure is observed in the cluster “A”, this clade consists of 28 haplotypes, the most frequent, H10, was found in eleven localities sampled in Central Panama and six in Eastern Panama. It also included Altos de Piedra and Coiba haplotypes from Western Panama. Cluster “B” is constituted by four haplotypes. Of these, H15 was collected from six sites in Central Panama and nine from Eastern Panama, with H23 being the most frequent [Figure 2A]. In cluster “C” we recorded four haplotypes exclusively of localities sampled from Western Panama, demonstrating strong divergence support by Bayesian analysis [complementary data]. Thus, the network is characterized by few mutational steps [Figure 2A].

On the other hand, analysis linked a total of 26 haplotypes of the EF α-1 region demonstrating a lack of genetic structure in the *Lu. gomezi* populations. The patterns of a star-like shape as similarly obtained with the mitochondrial region, showed haplotype H1 as the most frequent in the center and several haplotypes surrounding it with one and three mutational steps [Figure 2B].

The Bayesian phylogenetic tree for the CB3-NIN region showed three clusters with *a posteriori* probability of 0.99-1.0. Other minor clusters were supported by low *a posteriori* probability values. The cluster with a higher *a posteriori* probability corroborated the divergences obtained in the haplotype network, inferred by the median-joining method [Figure 2A and B]. The Bayesian inference of EF α-1 region did not show similitude either, this analysis performed for mitochondrial and nuclear genes are supplementary data attached as Additional files 1 and 2.

We found 50% of the haplotypes in the peridomestic areas of fragmented environments, 40% in rural environments, and 10% in forest environments. Although this information is additional and not the main focus of the research, the predominance of the haplotypes in these sites is important to highlight due to the anthropogenic impact on the landscape of Panama [Table 1].

### Demographic history population

The results of Tajima D test (CB3-NIN) were negative but not significant (−0.998; p > 0.01) that indicated neutrality deviation due to a demographic expansion or not neutral selection. Nevertheless, Fu´s Fs values were significantly negative (−19.68; p < 0.02) detecting that a population expanded in the past. The assessment of demographic change with EF α-1 region using both Tajima D and Fu´s Fs was significantly negative (−2.08; p < 0.01 and −13.65; p < 0.02) respectively.

Considering that Fu´s Fs are sensitive estimators for detecting demographic population expansion, we included the analysis of mismatch distribution for characterization of expansion detected with both genes. A bimodal curve was observed with CB3-NIN region and smoother curves with EF α-1 region. SSD value (0.011; p > 0.05) for CB3-NIN did not reject the sudden expansion model, while SSD value (0.049; p > 0.05) for EF α-1 region was observed. The Harpending´s raggedness index determined a good fit of data (Additional file 3). The time since expansion of the population was estimated to be approximately 4,223,825 years ago corresponding to the Pliocene Epoch.

## Discussion

The use of the mitochondrial and nuclear marker in ecology has provided enough information to allow contrast of the past population processes of species [54]. The high content of A-T (74%) of the mitochondrial gene analyzed in *Lu. gomezi* is similar to that detected in other sandfly species: *Phlebotomus papatasi* and *Lu. evansi*[30,55]. This suggests a high rate of substitutions as a result of natural selection, favorable or unfavorable to populations [56]. A higher proportion of G-C (53%) observed in the nuclear gene in *Lu*. g*omezi* was comparable to the proportion verified in *Anopheles gambiae* (45%) and *Culex pipiens* (63%), characteristically of intron region [57,58].

The high haplotype diversity detected among populations indicated a large polymorphism or gene pool of the *Lu. gomezi*. The existence of this polymorphism is important for the survival and adaptability, considering that the natural habitat of Phlebotominae sandflies is destroyed because of anthropogenic factors [20]. However, we observed discordance in the level of nucleotide diversity between CB3-NINI and EF α-1 sequences. This may be because the mitochondrial gene is highly variable due to an elevated mutation rate and inheritence maternally [59]. While the EF α-1 is an intron region of low copy number of protein coding highly conserved [41].

The result obtained from AMOVA showed mild genetic structure but greater genetic variation within populations. The explanation is that gene flow occurs between the different populations, probably caused by the flying capacity of the species increasing their migratory potential. Similar results were found from the analysis of population genetics of *Lu. longipalpis*, *Lu. intermedia* and *Lu. shannoni* over a distance between 8 to 15 km, which suggested that individuals from sandfly populations were capable of flying more than 57 m, as previously recorded for sandfly species [2,60-64]. Thus, we hypothesized that *Lu. gomezi* is able to disperse over long distances, which could be gradually helped by the winds (adults) or passive movement of eggs and larvae. Studies across distant localities using other molecular markers are necessary to define vector capacity to disperse.

Meanwhile, pairwise Fst-value demonstrated significant genetic differentiation with the CB3-NIN region of the populations from Western Panama and Central Panama. We think that this genetic differentiation observed is influenced by the central mountain range with altitudes of 2,468 to 3,475 m. The mountain range represents a geographic barrier restricting altitudinal distribution of *Lu. gomezi*, estimated between 175–300 meters high [65]. Similarly, strong genetic differentiation was found between *Anopheles albimanus* populations from Costa Rica and Panama, also suggesting that the Central American mountain range is a physical barrier that limited the gene pool of malaria vector mosquitoes [66,67].

On the other side, slight genetic differences were detected from CB3-NIN and EF α-1 regions, among the sandfly populations from Eastern Panama and Central Panama (separated by the Panama Canal basin) could be explained because of the variety of climate and vegetation in Panama. The climate of Panama is influenced by the Inter-tropical Convergence Zone, oscillating one year to other and from one region to another. This variation allowed three types of tropical climate: Afi (very humid), Ami (humid), Awi (dry), according to Köppen Climate Classification [68]. The sampling locations from Veraguas, Coclé, Colon, and Panama (west of the province of Panama) registered precipitations and temperatures between 3,001-3,500 mm and 22°C-24°C, respectively, while in the region of Eastern Panama, the amount of rain and the temperature reported varied between 1,000-1,800 mm and 26°C-27°C, respectively.

As a result, the type of tropical climate of Panama provides enough conditions to enable vegetation heterogeneity and refuge for a great diversity of species [69]. *Lu. gomezi* samples from Central Panama were collected from borderline locations of tropical forest vegetation of low lands. While the Eastern Panama populations were from areas of vegetation in regeneration with evident anthropogenic impact. Thus, both climate and vegetation defined different ecotypes that can restrict gene flow due to environmental gradient. Climate changes were also used by [70] to justify the genetic differences found among the Brazilian populations of *Lu. whitmani*.

The star-like shape of network haplotypes, both for CB3-NIN and EF α-1, reflected a rapid and recent expansion of *Lu. gomezi* populations. The haplotypes of CB3-NIN (H-10, H-15, H-23) and haplotype EF α-1 (H-1) are older alleles or ancestral haplotypes dispersed in the Isthmus. The network patterns in clusters A and B for the CB3-NIN region and network EF α-1 region could be explained due to the sandflies migration with their hosts, mainly mammals.

Several mammal species (Canidae, Procyonidae, Sciuridae, Didelphidae, Dasypodidade) that currently are sandfly hosts established themselves in Panama during the north–south migration that took place during the closure of the Isthmus between the Miocene-Pliocene epochs [71-73]. We suggest this event as causal of the dispersal of *Lu. gomezi* because fossil records from the Miocene showed the presence of sandflies in fragments of mammal hair and in the microstructure of bird plumages in the Dominican Republic, suggesting their relationship with several hosts and possibly their transportation mechanism [74].

The H6 of CB3-NIN region from Coiba National Park (Veraguas) probably derived from the H11 detected in the populations of Central Panama, which indicates a possible migration of *Lu. gomezi* from the continent. Coiba was a continental island separated from South America in the Eocene, laying next to the Isthmus of Panama due to the decrease in the sea level in the last glaciation. This could have enabled the migration of wild animals and later, when the sea level increased, Coiba was isolated [75,76]. These factors could have isolated this haplotype, and it is also evidence that wild hosts could help disperse these vectors.

The cluster B clearly showed the divergence in a west population (Bocas del Toro) and this strengthened significantly with the Fst-value [Table 4], we suppose that the population could have become geographically isolated. However other observations are necessary to establish phenotypic differences and corroborate this assumption.

The fragmentation of the forests on Panama is reported since pre-Columbian times [77,78]. The prevalence of 10 (CB3-NIN) and 1 (EF α-1) ancestral haplotypes all types of environment and the percent of haplotypes found in the fragment and rural environments could indicate the displacement of *Lu. gomezi* because of disturbed areas. However, it is necessary to establish the consequences of fragmentation forest in the population genetics by increasing sampling in local forests.

The neutrality tests Fu´s Fs results were significantly negative, and mismatch distribution statistic and network haplotype support the assumption of population expansion in the past. Moreover, high haplotype diversity but low nucleotide diversity in EF α- region also suggests population expansion.

Is it the fact that the closure of the Isthmus of Panama affected by climate change in the world was the main factor that enabled the establishment of several species in America, allowing for faunal exchanges between North and South [79]. An alternative explanation of the population expansion is that conditions later to closure of the isthmus may have been favorable to its adaptation to the suitable habitat and different ecological conditions in Panama territory. According to our estimated date the Pliocene climate was much cooler, allowing the expansion of plant and animal species into new habitats [80]. The colonization and adaptation into new habitat can be observed in the star-like shape of network haplotype.

## Conclusions

In conclusion, *Lu. gomezi* is a species with a higher genetic pool or variability and mild population structure, due to possible capacity migration and local adaptation to environmental changes or colonization potential. However the existence of geographic barriers, such as the central mountain range separates a subpopulation. The establishment of *Lu. gomezi* in the Isthmus of Panama could be interpreted by the exchange of mammals in the American continent, vegetation and climate conditions of the epoch. Their relevance as a carrier of cutaneous leishmaniasis in Panama is important for health schemes, especially if we consider that sandflies have a wide-spread geographic distribution in Panama, and this distribution is possibly associated to the landscape changes caused by deforestation [20]. In this study, mitochondrial and nuclear analysis has provided important information to allow assembles about genetic population and evolutionary history useful to understand the implications of different population genetic structures for cutaneous leishmaniasis epidemiology. Thereby, proposing new perspectives for the control of leishmaniasis vectors.

## Abbreviations

CL: Cutaneous leishmaniasis; CB3-NIN: Cytb gene, inter-genetic region IGS-1; tRNA-Ser: Inter-genetic region IGS-2, and start of the NADH1; EF α-1: Elongation factor alpha-1.

## Competing interests

The authors declare that they have no competing interests.

## Authors’ contributions

Conceived and designed the experiments: AVC, MGT. Performed the experiments: AVC. Analyzed the data: AVC. Contributed reagents/materials/analysis tools/specimens identifications: MGT, JDAF. Wrote the paper: AVC, JDAF and MGT. All authors read and approved the final version of the manuscript.

## Supplementary Material

Additional file 1 **Bayesian tree based on mitochondrial CB3-N1N of populations of *Lu. gomezi.***
Click here for file

Additional file 2 **Bayesian tree based on nuclear EF α-1 of populations of *Lu. gomezi.***
Click here for file

Additional file 3 **Mismatch distribution of *Lu. gomezi *** **species based on (A) mitochondrial sequence CB3-N1N and (B) nucleotide sequences EF α-1.** Click here for file
