# Supplementary figures and images for: Phylogeography of the Lutzomyia gomezi (Diptera: Phlebotominae) on the Panama Isthmus
Source: Parasit Vectors. 2014 Jan 8;7:9. doi: 10.1186/1756-3305-7-9 (PMC3892078; doi:10.1186/1756-3305-7-9)

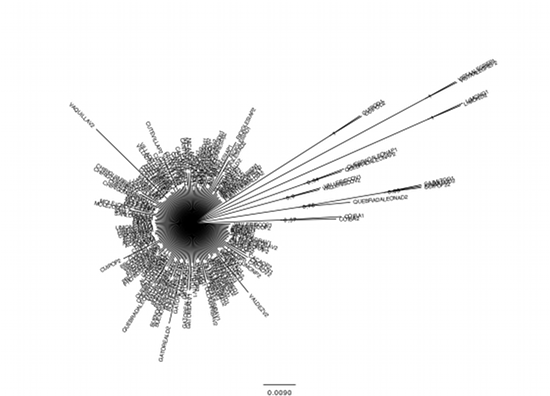

Supplement: Additional file 2 — Bayesian tree based on nuclear EF α-1 of populations of Lu. gomezi. [file 1756-3305-7-9-S2.tiff]

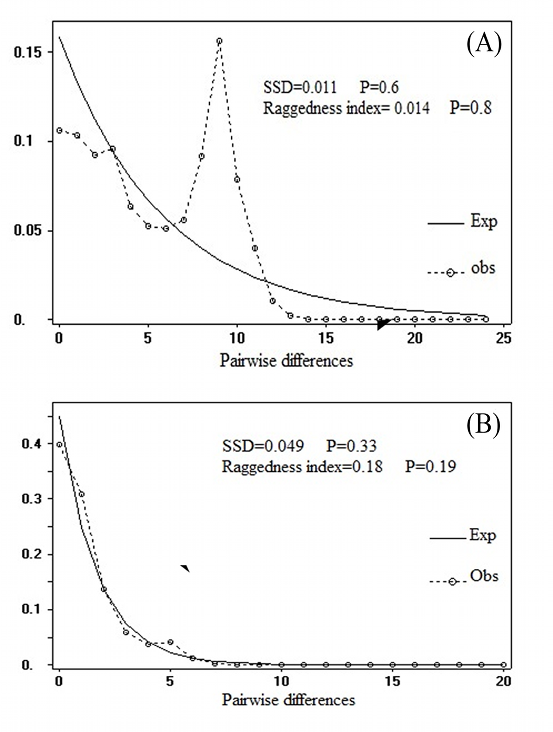

Supplement: Additional file 3 — Mismatch distribution of Lu. gomezi species based on (A) mitochondrial sequence CB3-N1N and (B) nucleotide sequences EF α-1. [file 1756-3305-7-9-S3.tiff]
