# Supplementary material for: Phylogeography of the Lutzomyia gomezi (Diptera: Phlebotominae) on the Panama Isthmus
Source: Parasit Vectors. 2014 Jan 8;7:9. doi: 10.1186/1756-3305-7-9 (PMC3892078; doi:10.1186/1756-3305-7-9)
Supplement: Additional file 1 — Bayesian tree based on mitochondrial CB3-N1N of populations of Lu. gomezi. [file 1756-3305-7-9-S1.pdf]

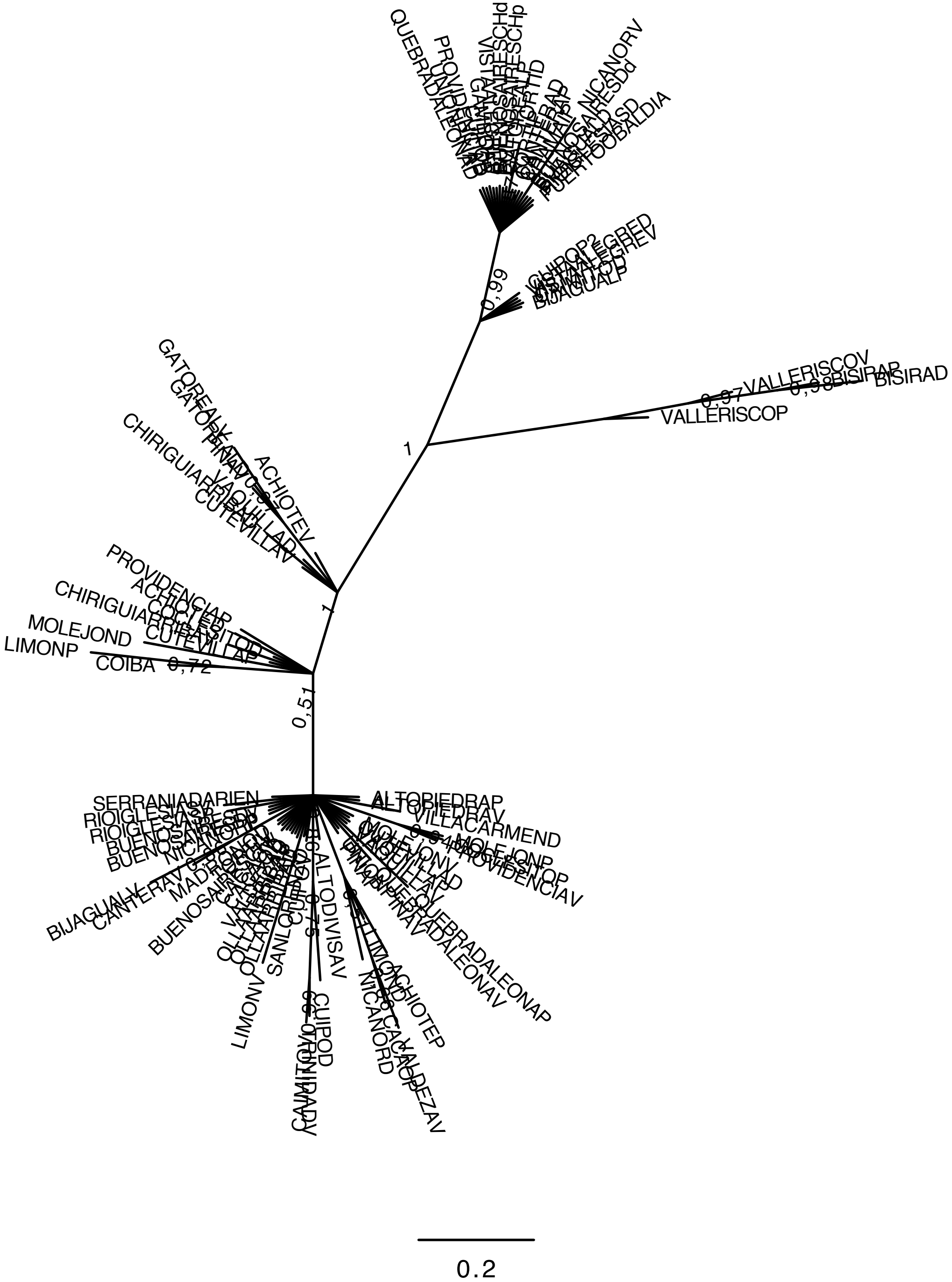

VALLERISCOV
BISIRAD
VALLERISCOP
PUERTOOBALDIA
BIJAGUALP
0,99
1
ACHIOTEV
PROVIDENCIAP
MOLEJOND
LIMONP
COIBA
0,72
0,51
SERRANIADARIEN
ALTOPIEDRAP
ALTOPIEDRAV
VILLACARMEND
PROVIDENCIAV
QUEBRADALEONAP
QUEBRADALEONAV
ALTODIVISAV
LIMONV
CUIPOD
NICANORD
ACHIOTEP
0.2
